# Rab3a Is Critical for Trapping Alpha-MSH Granules in the High Ca^2+^-Affinity Pool by Preventing Constitutive Exocytosis

**DOI:** 10.1371/journal.pone.0078883

**Published:** 2013-10-21

**Authors:** Simon Sedej, Maša Skelin Klemen, Oliver M. Schlüter, Marjan Slak Rupnik

**Affiliations:** 1 Division of Cardiology, Medical University of Graz, Graz, Austria; 2 Molecular Neurobiology and Neuroendocrinology, European Neuroscience Institute, Göttingen, Göttingen, Germany; 3 Institute of Physiology, Faculty of Medicine, University of Maribor, Maribor, Slovenia; 4 Molecular Physiology of the Brain, Göttingen Graduate School for Neurosciences and Molecular, Biosciences, Göttingen, Germany; 5 Centre for Open Innovations and Research, University of Maribor, Maribor, Slovenia; 6 Centre of Excellence for Integrated Approaches in Chemistry and Biology of Proteins, Ljubljana, Slovenia; UPR 3212 CNRS -Université de Strasbourg, France

## Abstract

Rab3a is a small GTPase of the Rab3 subfamily that acts during late stages of Ca^2+^-regulated exocytosis. Previous functional analysis in pituitary melanotrophs described Rab3a as a positive regulator of Ca^2+^-dependent exocytosis. However, the precise role of the Rab3a isoform on the kinetics and intracellular [Ca^2+^] sensitivity of regulated exocytosis, which may affect the availability of two major peptide hormones, α-melanocyte stimulating hormone (α-MSH) and β-endorphin in plasma, remain elusive. We employed Rab3a knock-out mice (Rab3a KO) to explore the secretory phenotype in melanotrophs from fresh pituitary tissue slices. High resolution capacitance measurements showed that Rab3a KO melanotrophs possessed impaired Ca^2+^-triggered secretory activity as compared to wild-type cells. The hampered secretion was associated with the absence of cAMP-guanine exchange factor II/ Epac2-dependent secretory component. This component has been attributed to high Ca^2+^-sensitive release-ready vesicles as determined by slow photo-release of caged Ca^2+^. Radioimmunoassay revealed that α-MSH, but not β-endorphin, was elevated in the plasma of Rab3a KO mice, indicating increased constitutive exocytosis of α-MSH. Increased constitutive secretion of α-MSH from incubated tissue slices was associated with reduced α-MSH cellular content in Rab3a-deficient pituitary cells. Viral re-expression of the Rab3a protein *in vitro* rescued the secretory phenotype of melanotrophs from Rab3a KO mice. In conclusion, we suggest that Rab3a deficiency promotes constitutive secretion and underlies selective impairment of Ca^2+^-dependent release of α-MSH.

## Introduction

Release of transmitters and peptide hormones from neurons and neuroendocrine cells into the extracellular space is triggered by cytosolic Ca^2+^ increase. Peptide hormones are packaged in specialized membrane compartments - secretory vesicles, which undergo a series of preparatory steps, including peptide expression, processing, maturation, storage and eventually Ca^2+^-dependent exocytosis. Dysregulation in vesicle biogenesis, docking, priming, and fusion may result in deficient or excessive peptide hormone release and the subsequent manifestation of neuro-/endocrine disorders.

Rab3 proteins are small regulatory GTPases expressed at high levels in most cell types that are specialized for secretion [1]. Rab3 isoforms (a-d) have been assigned heterogeneous functions during the life cycle of a secretory vesicle. These include the positive regulation of vesicle docking, priming, fusion and recycling [2-5]. Targeted deletion of Rab3a in mice does not appear to impair exocytosis *per se* and does not seem to be of crucial importance for nervous system function, since Rab3a-deficient mice are viable and do not show any apparent behavioral abnormalities [6]. In contrast, loss of Rab3a protein *in vitro* impairs the secretory output in the pituitary intermediate lobe cells – melanotrophs [7]. These cells primarily produce pro-opiomelanocortin-derived α-melanocyte stimulating hormone (α-MSH) and β-endorphin [8]. Rab3a stimulates secretory output at the step prior to exocytosis, possibly by recruiting vesicles containing a specific pituitary hormone [7]. However, it remains elusive whether Rab3a affects the kinetics and [Ca^2+^]_i_ sensitivity of the Ca^2+^-dependent secretory activity, and thus, the availability of the secreted pituitary hormones in plasma.

In neurons and neuroendocrine cells, such as mouse pituitary cells, Rab3a has been shown to form complexes with cAMP-guanine exchange factor II (GEFII)/Epac2 and Rim2 [9]. Yet, functional implications of Rab3a on the secretory response in pituitary at elevated cAMP have not been investigated. We previously demonstrated that cAMP positively regulates depolarisation-induced secretory response. Furthermore, we were able to discriminate between the GEFII/Epac2-dependent- and protein kinase A (PKA)-dependent component of the secretory response [10]. In this study, we aimed to determine whether Rab3a ablation affects specifically one of the aforementioned components. 

Here we have shown that ablation of Rab3a in mouse melanotrophs from pituitary tissue slices impairs the depolarisation-induced secretory activity due to the absence of GEFII/Epac2-dependent secretory component. The constitutive release of α-MSH was increased *in vivo* and *in vitro*, resulting in elevated plasma levels of α-MSH and reduced α-MSH cellular content in Rab3a KO pituitary gland, respectively. Overexpression of WT-Rab3a protein rescued both α-MSH expression and secretory phenotype *in vitro*. Our data suggest that α-MSH and β-endorphin release is differentially controlled by Rab3a, implying that release of vesicles with different content is regulated by different mechanisms. 

## Materials and Methods

### Mice

All animal procedures were performed in strict accordance with all national and European recommendations pertaining to work with experimental animals, and all efforts were made to minimize suffering of animals. The protocol was approved by the State of Lower Saxony, Germany, and the Veterinary Administration of the Republic of Slovenia (permit number: 34401-61-2009/2). Adult (age 8-20 weeks) Rab3a knock-out (Rab3a KO, N=29 mice) and their wild-type (WT, N=27 mice) littermates (control) were used for experiments [1,6,11]. PCR analysis of genomic DNA from ear/tail biopsies was performed for the assessment of genotypes. Mice were housed in a 12 h light/dark regime with access to food and water *ad libitum*. 

### Pituitary preparation

CO_2_-anaesthetized (2-min exposure) mice were sacrificed by cervical dislocation followed by decapitation. Pituitary glands were dissected carefully from the skull and pituitary slices were prepared for acute experiments or organotypic slice culture as described elsewhere [12]. Fresh slices (80 μm thickness) were transferred into an incubation beaker containing the oxygenated external solution 1 composed of the following (in mM): NaCl 125, KCl 2.5, NaH_2_PO_4_ 1.25, Na-pyruvate 2, myo-inositol 3, ascorbic acid 0.5, glucose 10, NaHCO_3_ 26, MgCl_2_ 3, CaCl_2_ 0.1, lactic acid 6. Slices were kept at 32°C up to 8 h. For the slice culture and viral infection, pituitary slices were transferred onto culture plate meshes (Millicell-CM, Millipore, Billerica, Mass., USA) inserted into a 6-well plate (Cellstar, Greiner Bio-One, Kremsmuenster, Austria). Slices were kept in an incubator at 37°C (95 % humidity and 5 % CO_2_) in a phenol red-free DMEM/F-12 medium (Life Technologies Inc., Grand Island, New York, USA) supplemented with 100 U penicillin and 100 μg ml^-1^ streptomycin medium before the experimentation.

### Immunocytochemistry

For immunostaining, pituitary slices were fixed in 4 % paraformaldehyde (PFA) in PBS, washed and permeabilized in 0.3 % Triton X-100 for 30 min. Remaining PFA and Triton X-100 were washed out with PBS for 10 min at room temperature. The cells were blocked by incubating the tissue slices for 30 min in PBS enriched with 3 % bovine serum albumin (BSA) and normal goat serum. Pituitary slices were then incubated with primary antibodies (rabbit anti-α-MSH antibody, dilution 1:200; Peninsula Laboratories, Inc., San Carlos, CA, USA and rabbit POMC/γ-MSH antibody, dilution 1:100; Phoenix Pharmaceuticals, Inc., Belmont, CA, USA) overnight at 4°C in blocking solution. Slices were washed in PBS and incubated for 45 min at 37°C with secondary antibodies (Alexa 488 anti-mouse antibody, dilution 1:500 or Alexa 647 anti-rabbit antibody, dilution 1:500, Invitrogen, USA) in PBS containing 3 % BSA. Pituitary tissue slices were mounted using Light Antifade (or Slowfade) Kit (Molecular Probes, USA). Confocal imaging was performed using a confocal microscope Leica DM IRE2 (Leica Microsystems, Germany) and the 488 nm Argon-ion and 633 nm He/Ne laser lines. Images were acquired and processed using the microscope software.

### Semliki forest infection of pituitary tissue slices

Semliki forest virus particles (SFV) were generated as described previously [13]. GFP-tagged plasmids for a Rab3aQ81L (GTPase deficient Rab3a mutant) or a Rab3aT36N (dominant-negative Rab3a mutant restricted to the GDP-bound form) or a WT-Rab3a encoding mutant were inserted into SFV. Multiple SFV dilutions were tested in pituitary slices to determine the optimal concentration and time required for the maximal infection rate. After allowing 4 h for infection, the virus containing phenol red-free DMEM/F-12 medium was replaced with a SFV-free extracellular solution 2 composed of the following (in mM): NaCl 125, KCl 2.5, NaH_2_PO_4_ 1.25, Na-pyruvate 2, myo-inositol 3, ascorbic acid 0.5, glucose 10, NaHCO_3_ 26, MgCl_2_ 1, CaCl_2_ 2, lactic acid 6. Slices were then directly transferred to a recording chamber for patch-clamp experiments. Cells expressing GFP-tagged Rab3a plasmids were identified using the excitation at 480 nm and patched within 8 h post infection. WT-Rab3a mutant expressed on a Rab3a KO or WT backgrounds (positive control) served as controls. 

### Radioimmunoassay

Blood was collected into EDTA-treated tubes (Sarstedt; Nümbrecht, Germany) and samples were centrifuged at 13200 rpm for 10 minutes at 4°C. Plasma (supernatant) was removed and stored at -20°C. Pituitary tissue slices (100 µm thickness) with comparable amounts of the intermediate lobe were collected into the ice-cold extracellular solution 2 and transferred onto Petri dishes containing the pituitary tissue culture medium [14]. For the quantification of constitutive hormonal release, slices (3 for each group) were incubated in a phenol red-free DMEM/F-12 medium for 24 h at 37°C. Medium samples were then collected, flash frozen in liquid nitrogen and stored at -80°C. The pituitary slices were further used for the quantification of hormonal cellular content (α-MSH and β-endorphin). Tissue probes were homogenized in the extracellular medium 2 and centrifuged at 13200 rpm for 1 min. Supernatant was collected and frozen at -80°C. For quantification of the KCl-triggered hormonal release, membrane depolarisation with 70 mM KCl for 1 min followed by a recovery in PBS for 1 min (cell repolarization) was repeated three times (3 slices per group). Both high KCl and PBS solutions containing secreted pituitary peptide hormones were collected. Samples were flash frozen in liquid nitrogen, stored at -80°C and combined to estimate the hormonal release during the cell membrane depolarisation. The radioimmunoassay analysis against α-MSH or β-endorphin from the samples was performed at IBL-Hamburg (Hamburg, Germany).

### Electrophysiology

Changes in cell membrane capacitance (ΔC_m_) and peak amplitude of high voltage-activated (HVA) Ca^2+^ currents were measured in single melanotrophs within the intact clusters of the intermediate lobe of pituitary gland [15] by using the conventional whole-cell patch-clamp configuration [16]. For HVA Ca^2+^ current and depolarisation-induced C_m_ measurements the slice was continuously perfused (1-2 ml min^-1^) with the extracellular solution 2. Patch pipettes were filled with the intracellular solution composed of (in mM): CsCl 140, Hepes 10, MgCl_2_ 2, TEA-Cl 20, Na_2_ATP 2, EGTA 0.05; pH was adjusted to 7.2 with CsOH. In a subset of experiments, cAMP (200 µM) was included into the pipette solution. At least 5 minutes were allowed for the cAMP effect [17,18]. Slow photo-release and cytosolic Ca^2+^ concentration ([Ca^2+^]_i_) measurements were performed as previously described [19]. For Ca^2+^- induced C_m_ measurements extracellular solution 3 containing (in mM); NaCl 125, KCl 2.5, NaHCO_3_ 26, Na_2_HPO_4_ 1.25, Na-pyruvate 2, ascorbic acid 0.25, myo-inositol 3, lactic acid 6, MgCl_2_ 1, CaCl_2_ 2 and glucose 3 was continuously supplied to the perfusion chamber. The pipette solution for Ca^2+^- induced C_m_ measurements was composed of (in mM): NP-EGTA 5, Fura-6F pentapotassium salt (both Invitrogen, USA) 0.01, CaCl_2_ 4, CsCl 125, Hepes 40, MgCl_2_ 2, TEA–Cl 20, Na_2_ATP 2; pH was adjusted to 7.2 with CsOH. Osmolality of extracellular solutions and intracellular solutions was 300±10 mosmkg^-1^, except for the experiments with hypertonic 500 mM sucrose. External solutions were continuously bubbled with carbogen (95% O_2_ and 5% CO_2_) maintain the pH at 7.3. All chemicals were purchased from Sigma (St. Louis, Mo., USA), unless otherwise stated. 

Cells were visualized with an upright microscope Nikon Eclipse E600 FN (Nikon, Tokyo, Japan) equipped with a CCD camera (Cohu, San Diego, CA, USA). Whole-cell currents and C_m_ changes (ΔC_m_) were measured with a lock-in patch-clamp amplifier (SWAM IIC, Celica, Ljubljana, Slovenia), low-pass filtered (3 kHz, -3 dB) and stored on a standard PC. Cells were voltage clamped at the membrane potential of -80 mV. Ca^2+^ currents were activated by 300 ms voltage ramps from -80 mV to 60 mV and leak corrected as described elsewhere [12]. For pulse generation, data acquisition and basic analysis we used WinWCP V3.3.3. software from J. Dempster (Strathclyde University, Glasgow, UK). High-resolution capacitance measurements (in the fF range) were made by using compensated technique as described previously [20], using a sinusoidal voltage (1600 Hz, 10 mV, peak-to-peak). Changes in C_m_ values were determined as described previously [10]. All electrophysiological experiments were performed at 30-32 °C. Signal processing and curve fitting were done using Sigmaplot (SPSS, Chicago, Illin., USA), Matlab (Mathworks, Novi, Mi., USA) and Matview (Wise Technologies, Ljubljana, Slovenia).

### Statistical analysis

Data are reported as mean±SEM. Differences between groups were tested by using Student’s *t*-test or one-way analysis of variance (ANOVA) followed by Tukey’s post-hoc test (SigmaSTAT, SPSS, Chicago, Illin., USA), when an overall significance was detected. The significance level was set at *P*<0.05. 

## Results

### Rab3a ablation impairs Ca^2+^-dependent exocytosis in melanotrophs

**Figure 1:**
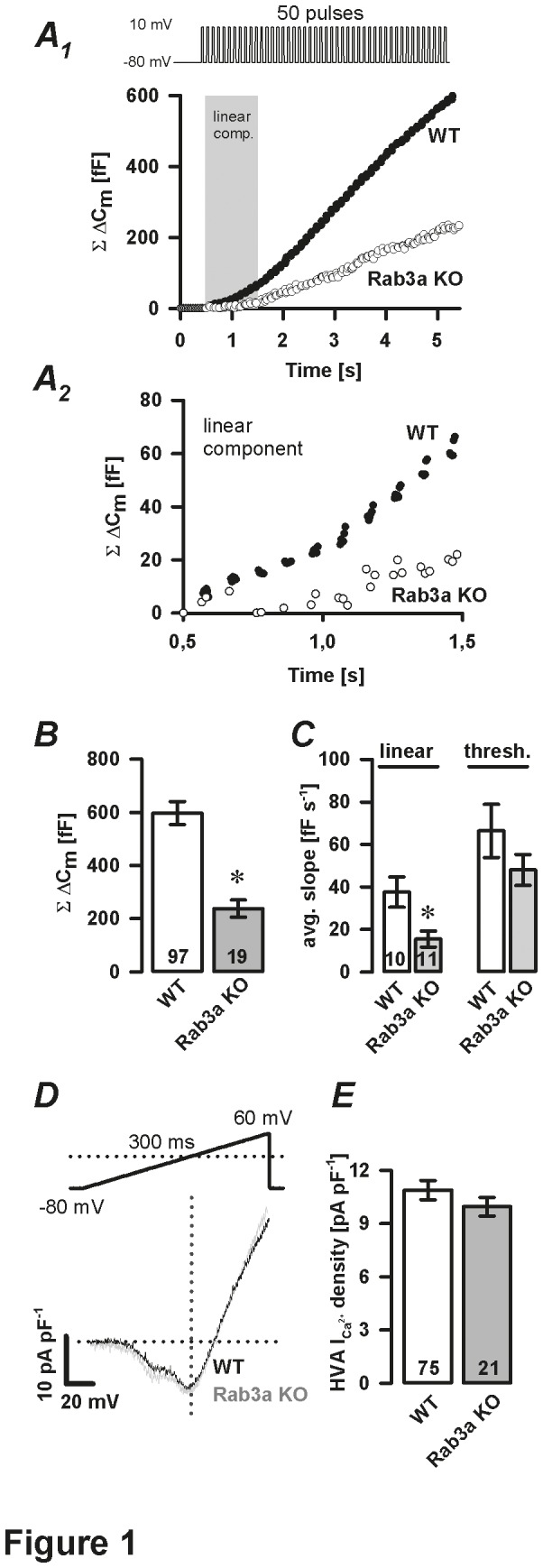
Depolarisation-induced secretory response in Rab3a KO melanotrophs. ***A*_*1*_**, Representative ΔC_m_ traces show Ca^2+^-dependent exocytosis triggered by a train of 50 depolarisation pulses (40 ms duration, at 10 Hz) in WT and Rab3a KO melanotrophs. Grey area confines the linear component (first second of depolarisation-***A*_*2*_**) from the subsequent threshold component. *B*, cumulative ΔC_m_ after 50 depolarisation pulses. *C*, summary of average slope of the linear component (grey shaded area-panel A_1_) and threshold component. *D*, normalized I-V plot. Voltage-activated Ca^2+^ currents evoked by 300 ms voltage ramps (from -80 mV to 60 mV) were normalized to resting membrane cell capacitance. *E*, High voltage-activated (HVA) Ca^2+^ current density. Numbers on bars indicate the number of tested cells. ^∗^ *P*<0.05 versus WT.

**Figure 2:**
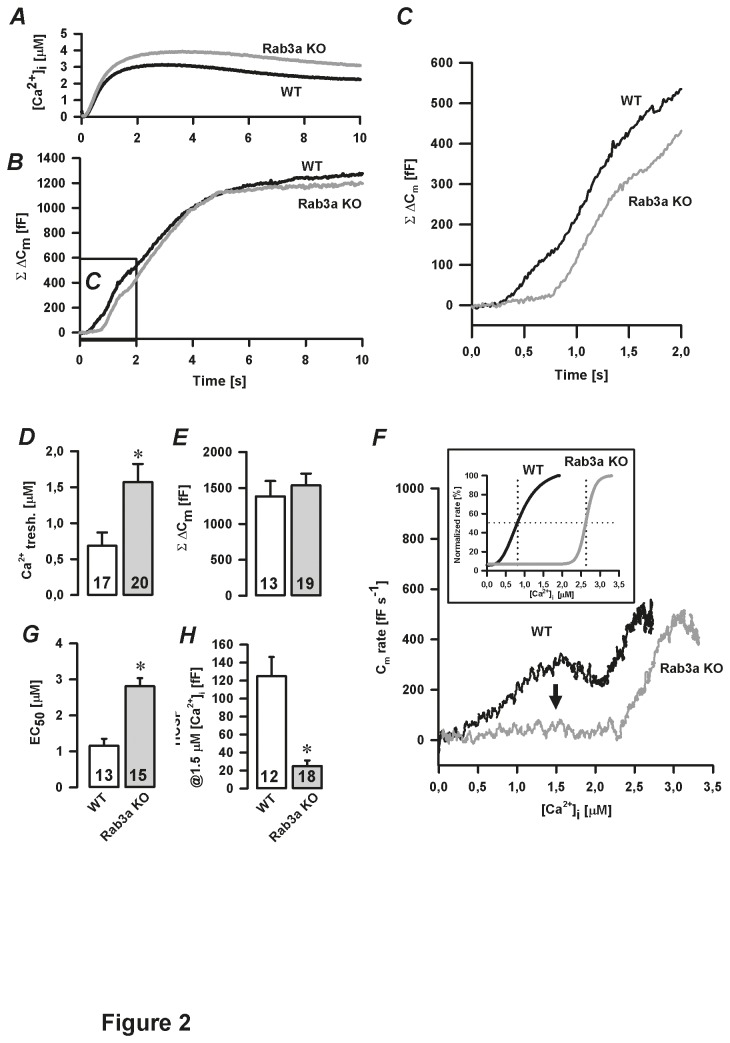
Initial high Ca^2+^ sensitive phase in Rab3a KO melanotrophs. *A*-*C*, slow photo-release of caged Ca^2+^ triggered a multiphasic ΔC_m_. The marked area is magnified in panel C. *D*, [Ca^2+^]_i_ threshold. *E*, cumulative ΔC_m_. *F*, time derivative of the ΔC_m_ presented in B as a function of [Ca^2+^]_i_ during the first 2 s of the slow photo-release of caged Ca^2+^. Note that high Ca^2+^ sensitive phase (vesicle pool) was greatly reduced in Rab3a KO cells (arrow), and that Ca^2+^ triggered ΔC_m_ at significantly higher [Ca^2+^]_i_ in Rab3a KO melanotrophs compared to WT cells (inset); the inset shows normalized C_m_ rate fitted by the Hill function and is displayed as a function of [Ca^2+^]_i_. *G*, half effective [Ca^2+^]_i_ (EC_50_). *H*, amplitude of high Ca^2+^ sensitive pool (HCSP) quantified at 1.5 µM [Ca^2+^]_i_ . Numbers on bars indicate the number of tested cells. ^∗^ *P*<0.05 versus WT.

We used whole-cell patch-clamp based capacitance (C_m_) measurements to assess the effect of Rab3a deficiency on the Ca^2+^-dependent exocytosis and its kinetics. Capacitance measurements indicate changes in membrane surface area that result from both exocytosis and endocytosis. In pituitary tissue slice preparations declines in C_m_ due to endocytosis following stimulus-induced rises in C_m_ are slow or absent [15]. Therefore, changes in C_m_ accurately reflect only the process of exocytosis. The Ca^2+^-dependent secretory activity was triggered using trains of depolarisation pulses (Figure 1A_1_, upper trace). The total C_m_ change (ΣΔC_m_) following 50 depolarisation pulses was significantly decreased in Rab3a-ablated cells (238±32 fF, n=19) compared to WT cells (597±44 fF, n=97, *P*<0.05, Figures 1A_1_-B). We previously showed that the depolarisation-induced change in C_m_ can be decomposed into two kinetic components: a first-linear component (ATP-independent) followed by a second-threshold component. The latter likely represents the exocytosis of release-ready vesicles and secretory vesicles that still require ATP-dependent reactions before fusion [10]. In Rab3a-ablated cells, the average slope of the linear component (during the first second of stimulation) of the ΔC_m_ was significantly reduced (15.5±3.8 fF s^-1^; n=11, *P*<0.05) compared to WT cells (32.6±5.0 fF s^-1^; n=18; Figures 1A_2_, C). On the other hand, the kinetics of the subsequent threshold component (WT: 66.4±12.6 fF s^-1^ versus Rab3a KO: 48.0±7.3 fF s^-1^, Figure 1C) as well as the HVA Ca^2+^ current density were comparable between the groups (Figures 1D-E). To more accurately determine the effect of Rab3a removal on the kinetics and [Ca^2+^]_i_ sensitivity of the Ca^2+^-dependent secretory activity, we used slow photo-release of caged Ca^2+^. This approach avoids relatively high variability in the Ca^2+^ current densities between individual cells measured by depolarisation stimuli [10]. In addition, slow photo-release allows an uniform increase in [Ca^2+^]_i_ throughout a cell during simultaneous ΔC_m_ measurements [19]. When [Ca^2+^]_i_ reached a certain level (Ca^2+^_tresh_, Figure 2A, D), a multiphasic ΔC_m_ was triggered (Figures 2B-C). At saturating [Ca^2+^]_i_, the cumulative C_m_ change was similar in both WT and Rab3a KO cells (1383±213 fF, n=13 versus 1537±163 fF, n=19, respectively; Figures 2B, E). Time derivative of the first two seconds of C_m_ traces distinguished one exocytotic component in Rab3a KO cells compared to two exocytotic components in WT cells (Figure 2F). The first Ca^2+^-dependent exocytotic phase was triggered at significantly higher [Ca^2+^]_i_ in Rab3a KO cells (1.8±0.3 µM, n=20, *P*<0.05) compared to WT cells (0.7±0.2 µM, n=17, Figures 2D, F). Normalized dC_m_/dt trace as a function of [Ca^2+^]_i_ was fitted using the Hill function. The average half-maximal rate of the C_m_ change (EC_50_) was achieved at 1.2±0.2 µM [Ca^2+^]_i_ in WT cells (Figure 2D inset and 2G; n=13). In Rab3a KO cells, however, EC_50_ was markedly increased (2.8±0.2 µM, n=15, *P*<0.05 versus WT, Figure 2G). A more detailed analysis revealed that the amplitude of the high Ca^2+^ sensitive phase (HCSP is defined by the vesicle fusion measured at 1.5 µM [Ca^2+^]_i_) was significantly reduced in Rab3a KO cells (25±6 fF, n=18) in respect to WT melanotrophs (125±21 fF, n=12, *P*<0.05, Figure 2H). Taken together, these results indicate impaired secretion of release-ready vesicles from the high Ca^2+^ sensitive pool in Rab3a KO melanotrophs. 

### Effect of cAMP on the secretory activity in Rab3a KO cells

**Figure 3:**
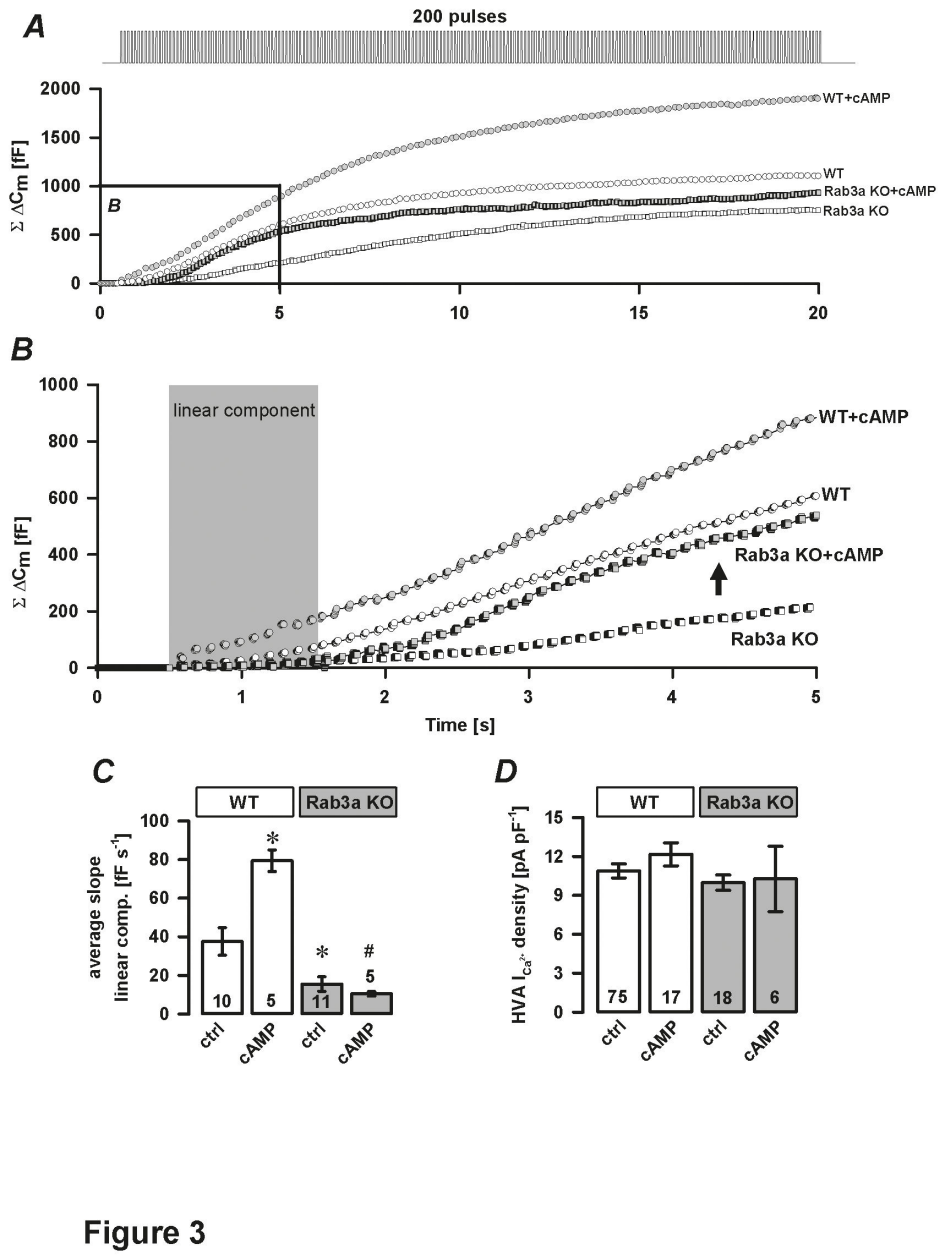
Effect of cAMP on Ca^2+^-dependent exocytosis in Rab3a KO melanotrophs. *A*-*B*, representative traces of total membrane capacitance changes (ΣΔC_m_) recorded during a train of 200 depolarisation pulses (40 ms duration, 10 Hz, from -80 mV to 10 mV) in WT and Rab3a KO cells treated without and with 200 μM cAMP in the pipette solution. The marked area is enlarged in panel B. Note that cAMP did not affect the linear component in Rab3a KO cells (grey area, enlarged inset). Note also a rapid increase in the ΣΔC_m_ as part of the subsequent threshold component (arrow) in Rab3a KO melanotrophs treated with 200 μM cAMP. *C*, average slope of the linear component. *D*, High voltage-activated Ca^2+^ current density. Numbers on bars indicate number of tested cells. ^∗^ *P*<0.05 versus WT control (ctrl). ^#^ *P*<0.05 versus cAMP-treated WT cells.

Because the linear component was impaired in Rab3a KO cells (Figure 1C), we tested whether high cAMP modulates Ca^2+^-dependent exocytosis via the threshold component (GEFII/Epac2-independent). Inclusion of cAMP in the pipette solution specifically increased the threshold (PKA-dependent) component in WT and Rab3a KO cells (Figures 3A-B). However, cAMP had no significant impact on the linear (GEFII/Epac2-dependent) component in Rab3a KO cells (10.5±1.2 fF s^-1^, n=5) compared to WT melanotrophs (79.4±5.6 fF s^-1^, n=5, *P*<0.05, Figures 3B-C). In addition, cAMP did not significantly alter the HVA Ca^2+^ current density in any of the groups studied (Figure 3D). To conclude, cAMP modulated the secretory function in Rab3a KO cells exclusively through PKA-dependent (threshold) pathway, indicating that Rab3a in WT cells acted likely via PKA-independent (cAMP-GEFII/Epac2-dependent) mechanism.

### Intact hypothalamic output in Rab3a KO mice

**Figure 4:**
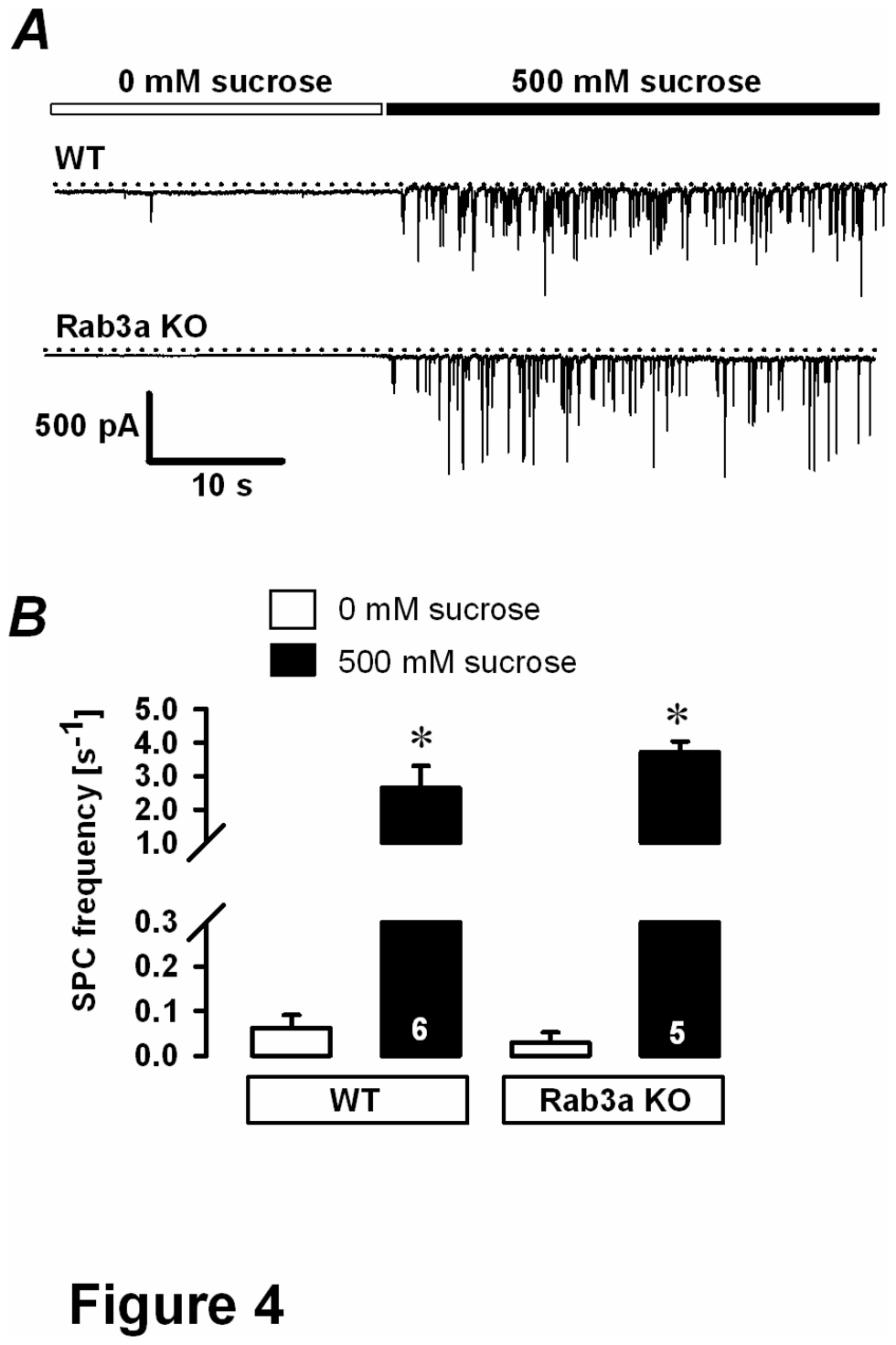
Effect of high sucrose in Rab3a KO cells. *A*, representative traces of spontaneous postsynaptic currents in melanotrophs from WT and Rab3a-ablated pituitary glands before and after sucrose (500 mM) administration. Cells were voltage-clamped at the membrane potential of -80 mV. Increased postsynaptic activity during 500 mM sucrose intervention implied an intact innervation from hypothalamic neurons. *B*, spontaneous postsynaptic current (SPC) frequency. Numbers on bars indicate number of tested cells. ^∗^ *P*<0.05 versus control (without sucrose).

The secretory defect in Rab3a-deficient pituitary cells might also be due to a dysfunctional release from hypothalamic terminals, for melanotrophs receive direct dopaminergic and GABA-ergic synaptic inputs [21,22]. Previous study on mouse pituitary showed a measurable GABAergic synaptic input that could be modified by addition of GABA or use of GABA_A_ antagonist, bicuculline [15]. Given that Rab3A has been defined to negatively regulate synaptic activity in neural preparation [23], a resulting overactivity could lead to hyperpolarized membrane potential on the target cell and changed excitability. To exclude this possibility, we inspected the innervation from hypothalamic neurons by recording the frequency of spontaneous postsynaptic currents (SPCs) in voltage-clamped melanotrophs during resting conditions and after the stimulation with hypertonic solution [24,25]. The addition of 500 mM sucrose significantly increased GABA release (Figure 4A) in both WT cells and Rab3a KO cells (WT: 2.64±0.66 Hz, n=6; Rab3a KO: 3.71±0.33 Hz, n=5; both *P*<0.05 versus 0 mM sucrose, Figure 4B), but these increases were not significantly different between the two groups. These results suggest that the lesion in the secretory activity in Rab3A KO mice can be primarily attributed to the pituitary cell and not to the change in hypothalamic inputs.

### Rab3a KO cells lack α-MSH, but not pro-opiomelanocortin

**Figure 5:**
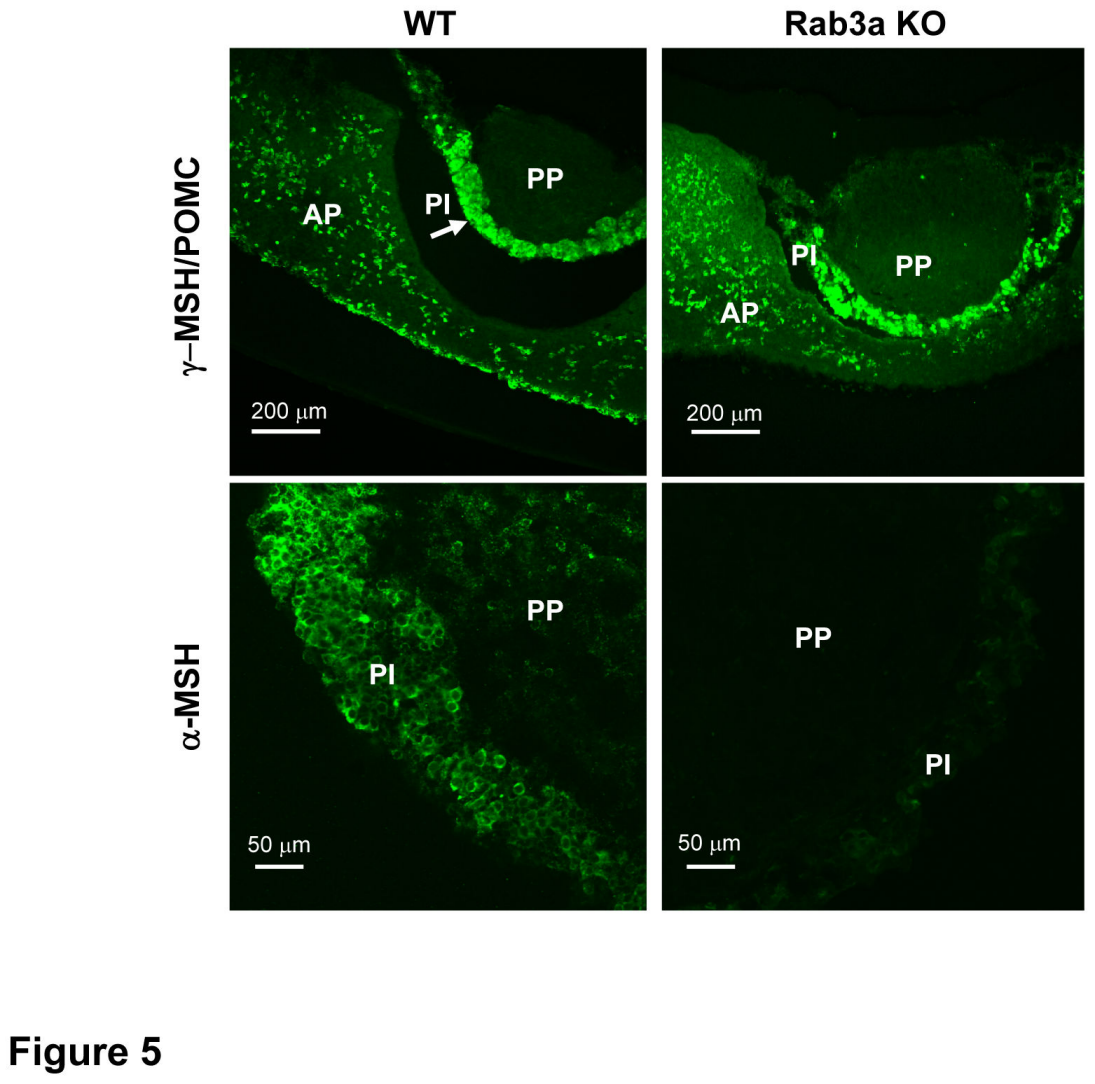
Alpha-MSH and pro-opiomelanocortin in Rab3a KO melanotrophs. Representative confocal microscopy micrographs from immunocytochemistry of γ-MSH/ pro-opiomelanocortin (POMC) (top) and α-MSH (bottom) in WT and Rab3a KO pituitary slices. Arrow indicates the intermediate lobe (PI). AP=anterior part, PP=posterior part. Rab3a KO melanotrophs contained POMC, but lacked α-MSH positive signal.

We then examined whether melanotrophs from Rab3a KO mice contain the appropriate peptide hormones, such as a predominant α-MSH and its precursor pro-opiomelanocortin (POMC). As shown in Figure 5, the expression of POMC from WT and Rab3a null mice was equally concentrated in the cells of intermediate lobe and some scattered cells in the anterior part of the gland as assessed by immunocytochemistry. Alpha-MSH appeared exclusively in the intermediate lobe of WT pituitary gland, while it was absent in the anterior lobe. Surprisingly, Rab3a KO melanotrophs almost completely lacked α-MSH, despite a strong POMC signal. 

### Impaired Ca^2+^-dependent α-MSH release from Rab3a KO melanotrophs

The absence of α-MSH immunoreactivity in POMC-rich Rab3a KO melanotrophs could result from impaired vesicle maturation or peptide conversion, but might also suggest the possibility that these two processes were intact and that the stability of mature vesicles containing α-MSH was reduced. First, the degradation of vesicles may be increased. Alternatively a formation of a standing pool of docked vesicles could be prevented. In the latter scenario, the secretory vesicles fuse immediately after maturation following the constitutive-like route.

**Figure 6:**
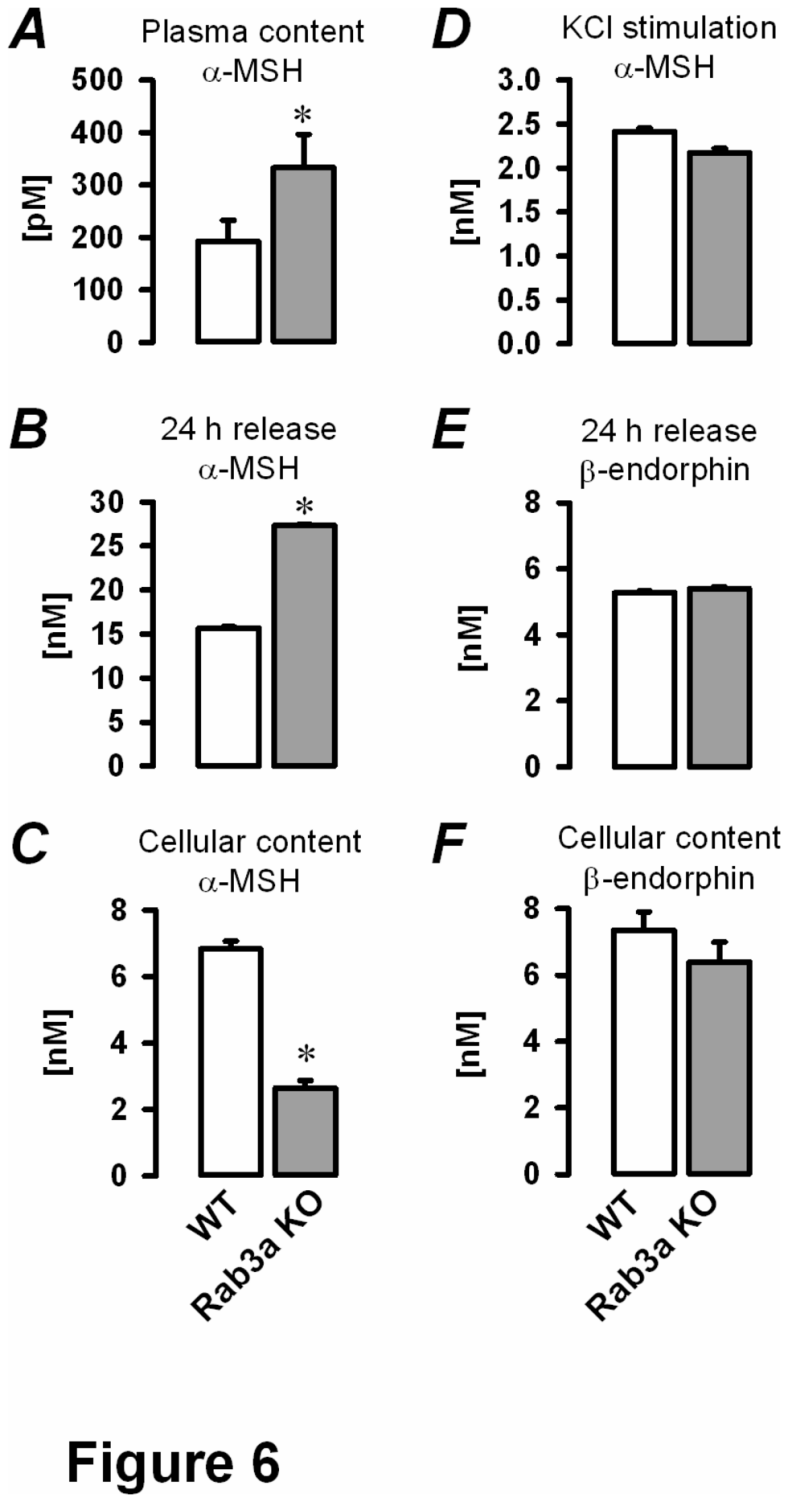
Constitutive and KCl-triggered release in Rab3a KO melanotrophs. Specimens from blood plasma and pituitary slices or pituitary tissue culture medium were subjected to the competitive radioimmunoassay. *A*, plasma concentration of α-MSH (N= 5 mice per group). *B*, constitutive release of α-MSH was measured from the pituitary tissue culture medium after 24 hours. Pituitary slices were then homogenized and ***C***, the cellular content of α-MSH was determined. *D*, KCl-stimulation (70 mM) provoked strong depolarisation of pituitary slices and triggered full secretory release of α-MSH from cellular stores. *E*, constitutive release of β-endorphin after 24 hours incubation at 37°C. Samples (pituitary slices) were collected and ***F***, the cellular content of β-endorphin was quantified. ^∗^ *P*<0.05 versus WT.

To determine whether the secretory malfunction of Rab3a KO melanotrophs is a consequence of an impaired vesicle biogenesis or an increased constitutive secretion or both, we quantified the secretion through different hormonal release pathways, including constitutive, regulated (triggered) and KCl-triggered hormonal release. Plasma concentrations of α-MSH and β-endorphin in WT and Rab3a KO mice, the respective release of both hormones from incubated pituitary tissue slices as well as the cellular content of were determined using radioimmunoassay. Alpha-MSH plasma levels were significantly increased in Rab3a KO mice as compared to WT (333±64 pM versus 193±40 pM, *P*<0.05, Figure 6A). Non-stimulated (constitutive) secretion of α-MSH from Rab3a KO cells was significantly elevated during 24 h incubation of tissue slices in the culture medium (27.33±0.17 nM versus 15.66±0.16 nM in WT cells, *P*<0.05, Figure 6B). Consistent with this, the cellular content of α-MSH was significantly reduced in Rab3a KO melanotrophs (2.65±0.23 nM) in respect with WT cells (6.83±0.24 nM, Figure 6C). The application of KCl triggered reduced Ca^2+^-dependent release of α-MSH from Rab3a KO slices (2.18±0.05 nM) compared to WT slices (2.41±0.04 nM), but this did not reach statistical significance (Figure 6D). In contrast to α-MSH, non-stimulated release and cellular content of β-endorphin were similar in WT and Rab3a KO pituitary (Figures 6E-F, respectively). These results in conjunction with the immunofluorescence imply that Rab3a deficiency promotes the constitutive exocytosis of α-MSH containing secretory vesicles in pituitary mouse melanotrophs.

#### Restored secretory function of Rab3a KO melanotrophs in vitro

**Figure 7:**
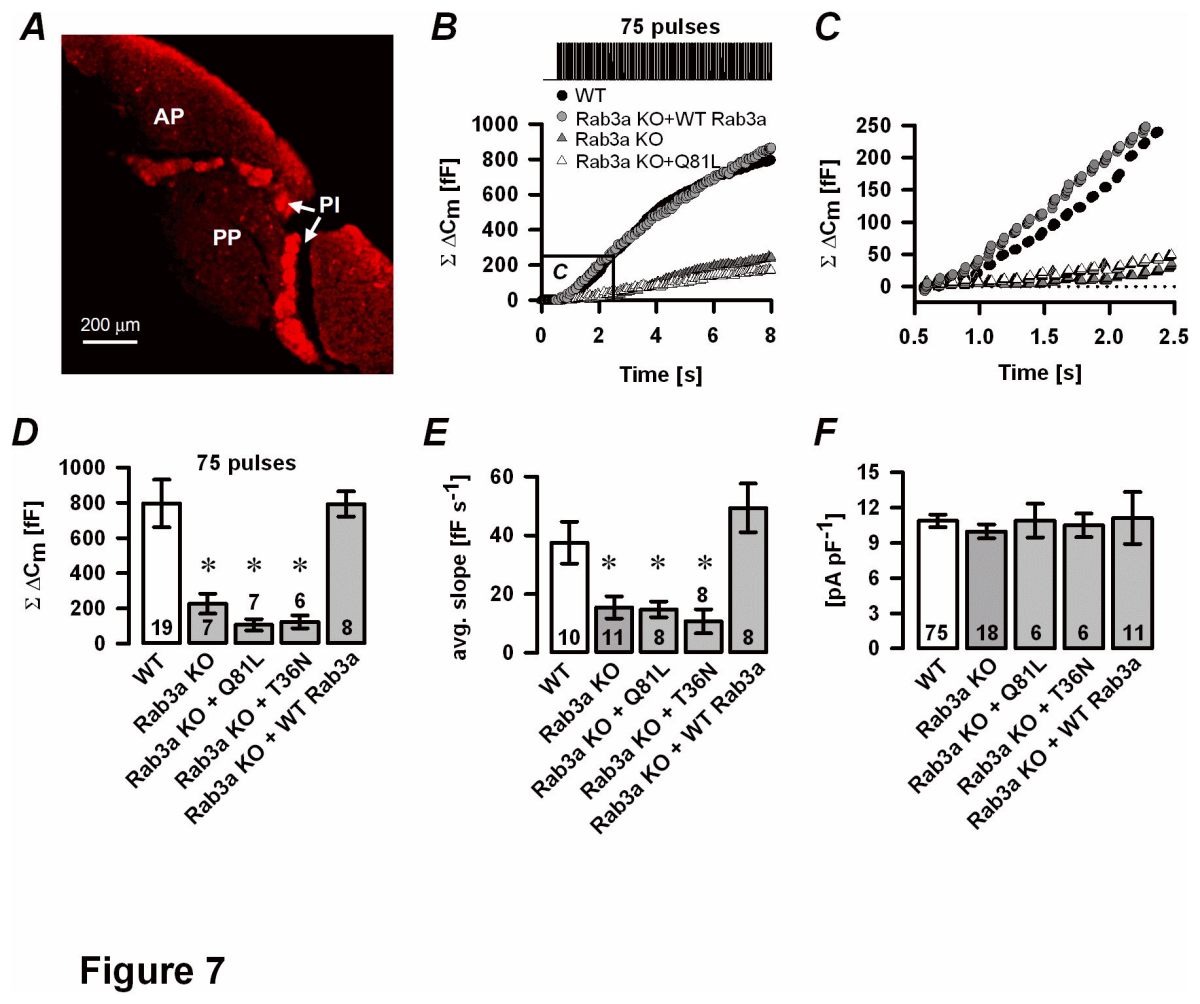
Rescue experiments in Rab3a KO melanotrophs. *A*, Semliki forest virus transduction of a WT-Rab3a plasmid restored α-MSH in Rab3a KO pituitary slice. *B*, Representative cumulative ΔC_m_ traces show depolarisation-induced secretory response in WT cells (black circles), Rab3a KO cells (grey triangles), Rab3a-deficient melanotrophs infected with Semliki forest virus harbouring a GTP-ase deficient Rab3a mutant (Rab3aQ81L, white triangles) or WT-Rab3a mutant (Rab3aKO+WT-Rab3a, grey circles). A repetitive stimulation of 75 pulses (40 ms stimulation, 10 Hz from -80 mV to 10 mV) evoked Ca^2+^ -dependent exocytosis. The marked area is magnified in panel C. *C*, The linear component was attenuated in Rab3aQ81L and Rab3a KO cells, but restored in Rab3a KO melanotrophs overexpressing a WT-Rab3a mutant. *D*, cumulative ΔC_m_. *E*, average slope of the linear component. *F*, High voltage-gated Ca^2+^ current density. Numbers on bars indicate the number of tested cells.∗P<0.05 versus WT.

To restore the secretory competence in Rab3a KO cells, we used the SFV-based short-term overexpression of a WT-Rab3a mutant on a Rab3a null background. GTPase-deficient Rab3a mutant (Rab3aQ81L) and a dominant-negative Rab3a mutant restricted to the GDP-bound form (Rab3aT36N) were used as controls. Rab3a KO melanotrophs infected with a WT-Rab3a mutant (Figure 7A) as well as Rab3aQ81L and Rab3aT36N (not shown) were positive against α-MSH. SFV-mediated transduction was effective enough to allow further electrophysiological analysis. The total C_m_ increase after repetitive stimulation (75 depolarisation pulses) was similar in Rab3a KO cells with an overexpressed WT-Rab3a mutant as compared with WT cells (793±71 fF, n=8 versus 797±135 fF, n=19, Figures 7B, D). On the other hand, melanotrophs harbouring the Rab3aQ81L mutation (107±33 fF, n=7) or a Rab3aT36N mutation (124±37 fF, n=6, both *P*<0.05) had significantly decreased cumulative C_m_ change as compared to Rab3a KO cells overexpressing WT-Rab3a. Average slope of the linear component in Rab3aQ81L- and Rab3aT36N-infected Rab3a KO cells (14.8±2.7 fF s^-1^ and 10.8±4.1 fF s^-1^, both n=8, respectively) was comparable to non-infected Rab3a KO cells (15.5±3.8 fF s^-1^, n=11, Figure 7E). The overexpression of a WT-Rab3a on a Rab3a KO background increased average slope of the linear (first) component (49.4±8.3 fF s^-1^, n=8, Figure 7E), consistent with a functional rescue after an acute expression of Rab3a. This result also indicates that the secretion phenotype in Rab3a deficient melanotrophs is not secondary due to the chronic absence of Rab3a, but rather a direct consequence of the absence of Rab3a function. Notably, SFV infection did not significantly alter the HVA Ca^2+^ current density in any of the groups (Figure 7F).

## Discussion

In our study we used pituitary tissue slice preparation from Rab3a KO mice as a model to assess the role of Rab3a protein in the late steps of stimulus-secretion coupling. Furthermore, we assessed the effect of Rab3a ablation on the global balance of predominant intermediate pituitary hormones. We have shown for the first time that the signal for the POMC derivative α-MSH was almost completely absent in Rab3a KO mice, despite preserved expression of the pro-hormone POMC in the intermediate lobe of the pituitary gland slice in WT and Rab3A KO. The absence of α-MSH was unlikely due to the impaired vesicle biogenesis or increased vesicle degradation, as α-MSH release did not significantly differ between WT and Rab3a KO cells in response to a strong depolarisation, such as KCl. However, it is important to point out that such a stringent stimulus is not physiological and that α-MSH release driven by action potentials is likely compromised in Rab3a KO melanotrophs. In support of this hypothesis, we found that Rab3a KO mice possessed increased constitutive release with increased plasma levels of α-MSH. We could reproduce the increased constitutive release in static Rab3a null pituitary slice incubations, which have been associated with reduced α-MSH cellular content. These results are opposite to the findings in PC12 neuroendocrine cells, where the overexpression of Rab3a was shown to convert the regulated secretory pathway into a Ca^2+^-independent constitutive pathway by depleting the readily releasable pool of vesicles [1]. 

Rab3a was shown as a negative regulator of secretory activity in chromaffin cells [23,26], whereas a positive regulator of neurotransmitter/hormone release in many other preparations, such as hippocampal neurons [6,27,28] and melanotrophs [7]. In the latter study, Rupnik and colleagues demonstrated that the injection of Rab3a antisense probe completely inhibits Ca^2+^-dependent secretion during the slow dialysis of Ca^2+^ into the cytosol of isolated rat melanotrophs. In the present work exocytosis from mouse melanotrophs in fresh pituitary slices was stimulated using two different methods, including a depolarisation train protocol and slow photo-release of caged Ca^2+^. In agreement with our previous study [7], we found that the secretory response triggered during the initial depolarisation-induced exocytosis or initial Ca^2+^ slow photo-release was diminished in Rab3a KO melanotrophs. In slow photo-release, significantly higher [Ca^2+^]_i_ was required to trigger a C_m_ change in Rab3a null melanotrophs compared to WT controls. Given that under physiological conditions [Ca^2+^]_i_ probably does not exceed 1-2 µM [15], it is likely that only the initial C_m_ change triggered by the slow photo-release of caged Ca^2+^ is physiologically relevant. We also showed that the changes in the secretory activity in Rab3A KO mice were primarily attributed to the pituitary cell and did not reflect increased hypothalamic GABAergic input, which might result in reduced excitability of the melanotrophs (Figure 4).

The increased constitutive exocytosis and reduced cellular content of α-MSH, together with the decreased initial C_m_ phase in Rab3a KO melanotrophs imply that Rab3a could be responsible for the maintenance of a standing pool of vesicles with a low threshold for Ca^2+^- triggered exocytosis. Some synaptic proteins, such as a neuronal isoform of SNAP25, were described to trap the primed vesicles in so called standing pools by increasing the priming rate and decreasing the depriming rate [29]. We hypothesize that high Ca^2+^-sensitive secretory vesicles fuse immediately after reaching the final stages of vesicle biogenesis in Rab3a ablated pituitary cells. The remaining vesicles are low Ca^2+^ -sensitive and fuse when [Ca^2+^]_i_ reaches higher levels. Our data suggest that Rab3a protein is responsible for retaining the α-MSH-containing secretory vesicles in a pool of functionally docked vesicles. These docked vesicles in turn shield the release sites for likely immature vesicles with distinct Ca^2+^ sensitivities and fusion kinetics. In support of this view, a recent study described significant morphological differences in docked vesicles in different endocrine cells comparing WT and Rab3 ablated mice [30]. Based on our findings from the radioimmunoassay and patch-clamp experiments, it is tempting to speculate that high and low Ca^2+^-sensitive secretory vesicles are associated with α-MSH and β-endorphin, respectively. However, this study did not rule out the possibility that vesicle fuse in Ca^2+^-independent (constitutive) manner [1]. 

Four Rab3 isoforms (Rab3a-d) are expressed in the mouse pituitary gland [1]. Previous study by Rupnik et al. [7] showed that rat melanotrophs contain equal amounts of Rab3a and Rab3b and suggested that only Rab3a is involved in Ca^2+^-dependent exocytosis. In Rab3a KO mice, however, the genetic deletion of Rab3a may potentially lead to an up-regulation or abnormal subcellular localization of other Rab3 isoforms (including Rab3b), which may contribute to the increased constitutive secretion in Rab3a KO melanotrophs. However, our preliminary results on melanotrophs lacking other Rab3 isoform or combinations of Rab3 isoforms exclude this possibility (data not shown). 

Rab3 proteins cycle between GTP-bound (active) and GDP-bound (inactive) state to act as molecular switches, specifically associated only with the resting secretory vesicles [1,31,32]. In previous studies, GTPase-deficient Rab3a mutant (Rab3aQ81L) was shown inhibitory for secretory activity in chromaffin cells [23,26,32], PC12 cells [1] and in *Aplysia* neurons [33], whereas the GDPase-deficient mutant showed no effect on secretory activity. Similarly, expression of WT-Rab3a inhibited hormone release [1,26,34,35]. We found that overexpression of different Rab3a recombinant proteins (Rab3aQ81L, Rab3aT36N and WT Rab3a) in Rab3a KO melanotrophs resulted in positive staining against α-MSH. This indicates that just the presence of Rab3a is enough for a formation of a standing pool of α-MSH containing vesicles in melanotrophs, independent of its activation state. However, the infection of Rab3a-deficient pituitary tissue slice with a GTPase-deficient Rab3a mutant and a mutant deficient in GTP binding resulted in secretory responses not significantly different from those in Rab3a KO cells. These results imply that only overexpression of a fully functional WT-Rab3a protein rescued the functional defect in Ca^2+^ -triggered exocytosis in Rab3a KO cells. Our results are in accordance with a study by Lin et al. [36] , suggesting that a fully functional Rab3a protein, capable of cycling between GTP-bound (active) and GDP-bound (inactive) state, is required for normal triggered secretory activity in mouse melanotrophs. It is important to note that our findings are partially inconsistent with the effect of Rab3a recombinant proteins expressed in isolated rat melanotrophs, where GTP-bound Rab3a stimulates Ca^2+^-dependent exocytosis, whereas the mutant favoring the GDP-bound state inhibits regulated secretion [7]. This divergence is likely explained by different time duration allowed for compensatory mechanisms in the single-cell knockout approach compared with genetically modified mice and by the different stimulation protocols (Ca^2+^ delivery by means of slow dialysis vs. repetitive depolarizing stimulation), which were shown responsible for the activity-dependent modulation of release [32]. Given different regulation of secretory activity by Rab3a in central synapses, adrenal chromaffin cells and pituitary cells, it remains to be determined whether differences in the effector molecules interacting with Rab3a account for this discrepancy.

Previously we demonstrated that the depolarisation-induced secretory response comprises of an ATP-independent (linear) component, mediated through a PKA-independent (cAMP-GEFII/Epac2-dependent) pathway, and a subsequent ATP -dependent component mediated via PKA [10]. Based on these findings, we tested whether an addition of cAMP ameliorated exocytotic activity in Rab3a-ablated cells. Cyclic AMP increased the Ca^2+^-dependent secretion in both WT and Rab3a KO melanotrophs. However, a more precise analysis revealed that only the PKA-mediated exocytotic component was increased. In contrast, cAMP did not increase the amplitude or the rate of the first ATP-independent cAMP-GEFII/Epac2-dependent exocytotic phase in Rab3a KO melanotrophs. This suggests that the standing pool associated with a low Ca^2+^ threshold for release is dependent on the interaction of Rab3a with Rim2 and GEFII. This is consistent with a previous study, which demonstrated that cAMP-GEFII/Epac2 promotes fusion of vesicles with the plasma membrane by forming a complex with Rim2 and Rab3, both proteins implicated in the late stages of exocytosis [37]. 

In summary, our data suggest that Rab3a is important for trapping the secretory vesicles into the high Ca^2+^-affinity pool of docked granules. The absence of a functional Rab3a protein underlies loss of mature secretory granules containing α-MSH through constitutive exocytosis, resulting in increased levels of α-MSH in the plasma and depleted cellular content. The initial phases of Ca^2+^-dependent secretion likely involve tripartite Rab3a/rabphilin/Rim complex that operates through an ATP-independent but cAMP-GEFII/Epac2-dependent mechanism. Overexpression of GTP- or GDP-dominant Rab3a mutants rescued the standing pool of α-MSH vesicles, but they failed to support Ca^2+^-dependent exocytosis, suggesting the association of Rab3a with other protein complexes during vesicle priming and fusion. 
